# LFA1 Activation: Insights from a Single-Molecule Approach

**DOI:** 10.3390/cells11111751

**Published:** 2022-05-26

**Authors:** Naoyuki Kondo, Yoshihiro Ueda, Tatsuo Kinashi

**Affiliations:** Department of Molecular Genetics, Institute of Biomedical Science, Kansai Medical University, Osaka 573-1010, Japan; kondonao@hirakata.kmu.ac.jp (N.K.); uedayos@hirakata.kmu.ac.jp (Y.U.)

**Keywords:** LFA1, Rap1, talin, kindlin-3

## Abstract

Integrin LFA1 is a cell adhesion receptor expressed exclusively in leukocytes, and plays crucial roles in lymphocyte trafficking, antigen recognition, and effector functions. Since the discovery that the adhesiveness of LFA1 can be dynamically changed upon stimulation, one challenge has been understanding how integrins are regulated by inside-out signaling coupled with macromolecular conformational changes, as well as ligand bindings that transduce signals from the extracellular domain to the cytoplasm in outside-in signaling. The small GTPase Rap1 and integrin adaptor proteins talin1 and kindlin-3 have been recognized as critical molecules for integrin activation. However, their cooperative regulation of integrin adhesiveness in lymphocytes requires further research. Recent advances in single-molecule imaging techniques have revealed dynamic molecular processes in real-time and provided insight into integrin activation in cellular environments. This review summarizes integrin regulation and discusses new findings regarding the bidirectionality of LFA1 activation and signaling processes in lymphocytes.

## 1. Introduction

Integrins are heterodimeric transmembrane adhesion receptors that regulate adhesion and migration. To date, 24 α/β integrin subunit pairs have been discovered and characterized by ligand specificities, cell-lineage specific expressions, and biological functions. LFA1 (αL/β2, CD11a/CD18) belongs to the β2 integrin family that is exclusively expressed in leukocytes. The clinical importance of β2 integrins is exemplified by a rare hereditary disease, leukocyte adhesion deficiency syndrome I (LAD-I), in which the amounts of β2 integrins, including LFA1(αLβ2), Mac1(αMβ2), and αXβ2, are severely diminished by germline mutations of the β2 gene [1]. Mutant leukocytes cannot migrate across inflamed venules. Consequently, patients with LAD-I suffer from recurrent infections, and can die at a young age without bone marrow transplantation in severe cases. In addition to pathological conditions, LFA1 plays important roles in immunosurveillance processes, including lymphocyte homing and antigen recognition, vital processes for acquired immunity. The α4 integrins (α4β1 and α4β7), which are expressed in lymphocytes and marginally in neutrophils, also play a key role in lymphocyte trafficking [2].

The adhesive activities of integrins are low in resting lymphocytes, and are quickly augmented when stimulated by chemokines and antigens. Since the first report that TCR crosslinking increases the adhesive activity of LFA1 to ICAM1 without changes in cell surface expression [3], the unique property of integrin adhesiveness regulated by intracellular signaling (inside-out signaling) has been a major topic within the study of integrin regulation in leukocytes and platelets; in additional studies, this led to the identification of a key intracellular signaling molecule, the small GTPase Rap1, as well as critical integrin-binding adaptor proteins, talin1 and kindlin-3, in leukocytes [2,4,5,6,7].

**Figure 1:**
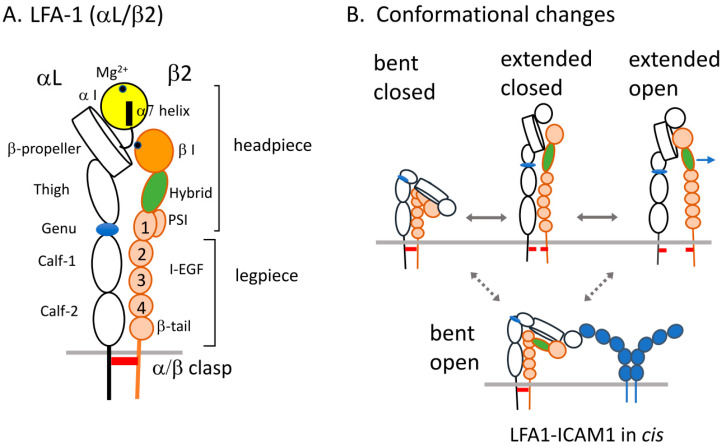
LFA1 structure and conformational changes. (**A**). LFA1 (αL/β2). The ectodomains of αL and β2 subunits are schematically shown in the extended conformation. The headpiece contains a ligand binding αI domain on top, β-propeller, and thigh domains in the αL subunit and regulatory βI and hybrid domains, and I-EGF1 in the β2 subunit. Mg^2+^ binding sites (dot) in αI and βI with the α7 helix and intrinsic ligand-containing loop are shown. The legpiece contains calf-1 and calf-2 in the αL and I-EGF2-4 in the β2. An inter-subunit association is depicted (α/β clasp). (**B**). Conformational changes of LFA1. Three classes of conformations are characterized by bending and extension of the ectodomain and headpiece closed-open conformations. The ectodomains are bent in a region (genu) between the thigh and calf-1 in the αL subunit, and between the I-EGF1 and I-EGF2 domains in the β2 subunit with a closed headpiece conformation. Upon stimulation or ligand binding, the bent-closed conformation is converted into extended-closed and extended-open conformations through positional changes of the hybrid domain (arrows representing hybrid domain swing-out into the open conformation). The bent-open conformation, discovered in slow rolling of neutrophils, is also shown.

Extensive studies of integrin structural changes related to affinity modulation have led to two major events in structural changes: the extension of the legpiece and the opening of the headpiece of integrins [8,9,10]. Upon stimulation and ligand binding, integrins globally rearrange conformations of the ectodomains from bent conformations to extended conformations with the closed or open headpiece (Figure 1). The liganded integrins transmit signals into the cells (outside-in signaling) to induce cell spreading and migration, and to affect cell growth and differentiation. In general, cell adhesiveness is a product of affinity (strength of the integrin–ligand bond), avidity (valency or clustering of integrins), and contact area. Thus, integrin adhesiveness is regulated by affinity and avidity, which vary based on the amount of integrins and ligands, as well as the time windows in which adhesive response proceeds in pathophysiological contexts. The introduction of sophisticated imaging, molecular, and biochemical methods has shed light on integrin regulation in situ. The key issue to be addressed is how integrin occupancy with adaptor proteins and ligand binding (i.e., bidirectional signaling) cooperatively regulates dynamic adhesion responses of lymphocytes in diverse microenvironments in the immune system.

## 2. Structure of LFA1 and Conformational Changes

Integrins are heterodimeric type I transmembrane receptors composed of α and β subunits. The ectodomains of integrins are assembled into a globular headpiece mounted on top of a legpiece, followed by a transmembrane and short cytoplasmic region. The headpiece of LFA1 is composed of the α-subunit αI, β-propeller, and thigh (upper leg) domains, and the β-subunit βI, hybrid, and PSI/I-EGF1(upper legs) domains. The lower legs are the α subunit calf-1 and calf-2 domains, and the β subunit I-EGF1-4 and β tail domains [9] (Figure 1). The αI domain contains a ligand-binding metal ion-dependent adhesion site (MIDAS) on top that binds Mg^2+^ ion to coordinate a glutamic acid residue in domain 1 of ICAM1 [11], a major ligand of ICAMs expressed on a wide variety of cell types, including leukocytes, endothelial cells, and dendritic cells [12,13]. In the case of integrins lacking the αI domain, such as α4, αIIb, and αV integrins, the βI domain containing the MIDAS and a part of the β-propeller together form a ligand binding site [14,15,16]. The βI domain also contains a site adjacent to MIDAS (ADMIDAS) that binds to the inhibitory Ca^2+^ ion, which competes with the activation of the Mn^2+^ ion [17,18].

Conformation and affinity changes of integrins including β1, β2, and β3 have been extensively investigated, revealing an association between ligand-binding affinity and distinct conformations. Inactive integrins exhibit the bent form of the ectodomains with low ligand-binding affinity, and undergo two types of conformational changes upon activation and ligand binding: switchblade-like legpiece extension, and opening the headpiece with the hybrid domain swing-out [9,19,20,21]. The extension and bending movements of the ectodomains occur at the genu between the thigh and calf-1 of the α subunit, and between I-EGF1 and I-EGF2 of the β subunit. The αI domain engaged with ICAM1 displaces the α7 helix downward, which makes a glutamic acid residue in the linker region available as an internal ligand to bind the βI MIDAS, leading to the hybrid domain swing-out and separation of the legpiece and subsequent integrin activation [9] (Figure 1). Thus, integrin conformations in resting states are predominantly in bent-closed conformations with low ligand-binding affinity, whereas inside-out activation and ligand binding shift the equilibrium toward extended-closed conformers with an intermediate affinity state and extended-open conformers with high affinity [9,22,23].

The bent-closed conformations are stabilized by inter-subunit associations (clasp) involving the cytoplasmic tails and transmembrane domains [24]. Loosening of the α/β associations by disruption of an inhibitory salt bridge via mutations of the conserved GFFKR motif in the α subunit and corresponding acidic amino acids in the β2 subunit expose neoepitopes in the legpiece and βI domain, and induce adhesion [25]. Indeed, the stimulation of lymphocytes with chemokines or adhesion to ICAM1 in the presence of manganese has been shown to induce separation of the cytoplasmic tails of LFA1 detected by the FRET (Fluorescence Resonance Energy Transfer) between the C-terminal ends of the αL and β2 subunits, suggesting that the unclasping of α/β subunits is actually induced by inside-out and outside-in signaling [26]. Similarly, FRET analysis of the ligand-mimetic peptide bound to VLA-4 and the plasma membrane suggests that VLA-4 is extended [27]. In addition, reconstituted full-length αIIbβ3 embedded in lipid nanodiscs exhibits a significant increase in extended conformations by the talin head [28]. Because the ligand-binding domains in the bent form point toward the plasma membrane, the extension is critical for macromolecule ligands, such as intact extracellular matrix and counterreceptor ICAMs, but not for soluble small ligands.

It is currently unknown to what extent the ectodomain extension can be induced from the inside of the cells, and to what extent it is required for adhesion. The conformational changes appear to be different in integrin species. The αXβ2 ectodomain has shown to be more resistant to unbending than that of LFA1 [20]. Consistently, activating and reporter antibody epitopes in the β2 legpiece of αXβ2 are exposed to a lesser degree compared to those in LFA1 and Mac1 [29]. Interestingly, the bent-open conformers of LFA1 have been detected in slow neutrophils rolling along the endothelium using reporter antibodies and high-resolution quantitative dynamic footprinting microscopy [30] (Figure 1B). In this case, bent-open LFA1 conformers bind to ICAM1 dimers on the same plasma membrane (*cis*) face-to-face, rather than on the apposed membrane of the endothelium (*trans*). The *cis* interactions of LFA1 and ICAM1 inhibit arrest and adhesion in neutrophils [30,31]. The alternative transition pathway from bent-open conformers to extended-open conformers is crucial to allow binding to ICAM1 on the endothelium. In the rolling under shear flow, chemokine-induced partial extensions with flexible αI domain orientations relative to the β propeller and βI domains [21] may allow LFA1 to bind to ICAM1 in the *cis* conformation.

## 3. Intracellular Regulators of LFA1 Activation

### 3.1. Rap1

**Figure 2:**
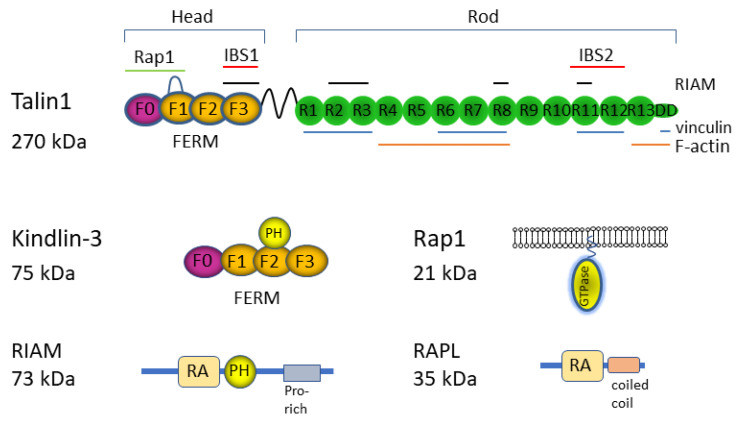
Integrin adaptors and Rap1 signaling molecules. Integrin adaptor proteins (talin1 and kindlin-3), Rap1, and Rap1 effector molecules are depicted with domain composition (see text). F0–F3 indicate subdomains of 4.1, ezrin, radixin, moesin (FERM) domain in Talin1 and Kindlin-3. R1–R13 indicate subdomains of ROD domain in Talin1. DD indicates dimerization domain. PH indicates pleckstrin homology domain. RA indicates Ras-associating domain.

Activated Rap1 is a potent stimulator of integrin adhesiveness that mediates inside-out signaling downstream of antigen and chemokine receptors through various effector molecules [4,32] (Figure 2). Rap1 has two highly homologous isoforms, Rap1a and Rap1b, which have overlapping integrin functions. Both Rap1a and Rap1b are expressed in hematopoietic cells, with greater amounts of Rap1b in platelets [33], neutrophils [34], B cells [34], and T cells [35]. Knockout of both *Rap1a* and *Rap1b* genes in T cells severely impaired chemokine- and TCR-induced adhesion and immunological synapse (IS) formation [35,36,37,38]. Rap1, like other small GTPases, operates as a binary switch between the GDP-bound inactive state and the GTP-bound active state, regulated by activating guanine exchange factors (GEF) and inactivating GTPase-activating proteins (GAP). Among many GEFs and GAPs, RasGRP2 (Cal-DAG-GEFI) [39], C3G (RapGEFI) [40,41], and Rasa3 [42] regulate Rap1 functions for integrin in leukocytes and platelets. Rap1, modified with a geranylgeranyl lipid moiety at a cysteine of the C-terminal CAAX motif, localizes in vesicular compartments, the Golgi complex, and the plasma membrane [32]. TCR-induced Rap1 activation occurs in these locations in T cells [35]. GTP-bound Rap1 binds to effector molecules to exert biological functions. Currently, there are two major Rap1 effector molecules in T cells, RAPL [43] and RIAM [44], which bind to Rap1-GTP. RAPL and RIAM involve Rap1 signaling to kindlin-3 and talin1, respectively, as described below. In addition, Rap1 has shown to directly bind to talin1 through the N-terminal regions of talin1 (F0 and F1 subdomains) [45,46,47]. Although the interaction of soluble Rap1-GTP and the F0 subdomain is weak (*K*_D_~0.16 mM), membrane-anchored Rap1-GTP can bind to an N-terminal head of talin1 with 100-fold higher affinity [46]. Indeed, the mutations of the F0 domains of talin1 impair neutrophil and platelet integrins variably depending on the mutation sites [48,49]. Thus, talin1 is a direct Rap1-binding effector.

### 3.2. Talin1

Talin associates with the cytoplasmic tail of β subunits through the proximal NPxY/F motif, and links to the F-actin cytoskeleton to loosen α/β clasps, thereby relaying a conformational change of the cytoplasmic tails to the transmembrane and ectodomains [6]. Therefore, talin1 translocation to the plasma membrane and association with β-tails is thought to be a critical event in the final stage of integrin activation by inside-out signaling [50,51]. The absence or inactivation of talin1 in embryos is lethal, and lineage-specific deletion of talin1 causes severe defects in platelet and lymphocyte integrins [37,52,53]. Talin1 is a dimeric cytosolic protein with an N-terminal head (talin-H) and long rod (talin-R) regions (Figure 2). Talin-H contains the atypical FERM domain composed of F0, F1, F2, and F3 subdomains, and talin-R contains 13 helical bundle domains (R1–R13) [6]. The F3 subdomain of talin-H binds to the proximal NPxY/F motif (integrin-binding site 1; IBS1) and preceding amino acids, as well as phosphatidylinositol-4-phosphate 5-kinase type Iγ (PIP5Kiγ) and Rap1 effector RIAM [54,55]. Talin-R interacts with F-actin, vinculin, RIAM, and various cytoplasmic components [6]. The rod R11/R12 constitutes the second integrin-binding site (IBS2) that binds to the conserved glutamic acids in the membrane proximal region of the β subunit [56].

Structural studies have revealed the existence of an autoinhibited compact talin1 dimer through intramolecular associations between F3 and R9, masking the integrin-binding site of F3 [57,58]. Negatively charged membrane lipids, such as PIP2, tether and activate talin via association with positively-charged residue of the F2–F3 of talin-H, while repelling talin-R by an electrostatic push–pull process [59]. Although the ablation of PIP2-producing PIP5KIγ had modest impacts on the talin-mediated adhesion [60], single or double knockout of PIP5KIγ and Rap1 has shown that PIP5KIγ and Rap1 cooperatively regulate the neutrophil slow rolling and arrest that require talin1 [61]. This work supports the notion that Rap1 binding to the talin F0–F1 domains works synergistically with membrane lipid PIP2 binding via the F2–F3 domains to recruit talin to the membrane [45], thereby facilitating talin association with the membrane proximal NPxY/F motif of the β tail.

### 3.3. Kindlin-3

Kindlin-3 is another key integrin-binding adaptor protein, mutations of which cause LAD type III [62,63,64,65]. Kindlin-3 is a member of the kindlin family (kindlin-1/-2/-3) that is exclusively expressed in hematopoietic cells [7]. The mutations of kindlin-3 severely impair the adhesiveness of β1, β2, and β3 integrins in leukocytes and platelets, leading to recurrent infections and bleeding disorders. Kindlins are cytosolic proteins containing an atypical FERM domain with a pleckstrin homology (PH) domain insertion in an F2 subdomain (Figure 2). The F3 subdomain of kindlin-3 binds to a distal NPxY/F motif and preceding threonine residues of the β subunit. Kindlin-3 has been shown to be involved in the affinity modulation of LFA1 in neutrophils [66]. The regulatory models of integrin activation involve a coordinated regulation of kindlins as a coactivator for talin, or consecutive interactions of kindlins and talin with β tails to recruit activators, repel inhibitors, or induce integrin clustering [5,6,51]. Currently, it is not fully understood how kindlin-3 contributes to LFA1 activation due to minimal evidence of direct interactions between talin and kindlins [67]. Structural and functional studies of kindlins have shown that the PH domain of kindlin-3 is essential for integrin-dependent adhesion, and has binding affinity for PI(3,4,5)P3 over PI(4,5)P2 [68], which may stabilize the membrane association of kindlin-3 in rolling neutrophils [69]. In addition, the PH domain of kindlins directly associates with paxillin [70] in a mutually exclusive manner with PIPs [71]. Moreover, the F0 subdomain in kindlin-1 and kindlin-2 also binds to paxillin. The paxillin binding to kindlins is important for focal adhesion assembly and cell spreading in fibroblasts, CHO cells [72,73,74], and platelets [71]. Kindlins are also known to interact with various proteins [7], including F-actin [75]. Thus, kindlins may sequentially bind to integrins and cytoskeletal proteins involved in post-adhesion events, thereby playing additional roles in integrin avidity regulation [76]. Further structural studies have shown oligomer formation of kindlins, which affects integrin activation positively or negatively [77,78,79]. Although the truncated mutants of kindlin-2 and kindlin-3 lacking the PH domain and the loop in F1 subdomain are able to form a dimer [77,78], full-length kindlin-3 and kindlin-2 exhibit an auto-inhibitory homotrimer formation in addition to the monomeric, but not dimeric form [79]. In the kindlin-3 trimer, the integrin-binding pocket of the F3 subdomain in one kindlin-3 is occluded by the PH domain in another, inhibiting their binding to the β1 integrin tail. Consistently, a mutant kindlin-3 with defective trimer formation increases adhesion to fibronectin [79]. It would be interesting to determine the mechanism of association and dissociation of kindlins in integrin activation processes.

### 3.4. RIAM

RIAM, a member of the Mig-10/RIAM/lamellipodin (MRL) family (Figure 2), has a Rap1-GTP-binding RA domain and a PIP2(4,5)-binding PH domain. The N-terminal region of RIAM can bind to the talin head, F3, to release autoinhibition of talin1, and facilitate membrane association of talin-H [44,80,81]. Thus, RIAM is thought to play an important role in the recruitment of talin1 to αIIbβ3 for activation [82]. Unexpectedly, RIAM-deficient mice have illustrated that RIAM plays a crucial role in myeloid cells and lymphocytes [37,83], but has no impact on αIIbβ3 and platelet functions [84]. RIAM deficiency causes the impairment of LFA1/ICAM1 adhesion in T cells and B cells upon stimulation with chemokines and antigen receptor crosslinking. On the other hand, β1 integrins are almost intact with defective attachment to ICAM1 in RIAM-deficient myeloid cells [37,83]. Furthermore, CD4^+^ regulatory T cells (Treg) lacking RIAM have intact functions of integrin adhesive activities, including LFA1, α4β1, and α4β7, despite strict requirements of Rap1 and talin1 for adhesion by these integrins and the suppression functions of Treg [85]. Thus, RIAM functions are critical for LFA1 in conventional lymphocytes, but not Treg. However, these results should be interpreted with caution, as there seems to be functional redundancy among MRL family proteins: Lamaellipodin (Lpd) compensates for the absence of RIAM in Treg to mediate adhesion by LFA1, VLA-4, and α4β7, as double knockout of genes encoding RIAM and Lpd severely impair integrin functions in Treg [85]. It is currently unknown whether Lpd can bind to and recruit talin1 to the plasma membrane in response to agonist-induced Rap1 activation. Although both Lpd and RIAM are actin polymerizing Ena/VASP ligands, the previous study ruled out the requisite involvement of RIAM-associated Ena/VASP proteins in adhesion [44]. Since functional differences and distinct specificities of RA and PH domains also exist [86,87], the relative contributions of different MRL proteins and regulatory mechanisms for integrin activation warrant further studies.

### 3.5. RAPL

RAPL (Rassf5c) is a member of the Rassf family [88], and is predominantly expressed in lymphocytes [43] (Figure 2). RAPL has a central RA domain that binds to Rap1-GTP, and a C-terminal coiled-coil region that binds to Mst1/Mst2 kinases [89]. The N-terminal region of RAPL interacts with the cytoplasmic tail of the αL subunit. RAPL activates Mst1, and thereby modulates both the affinity [35] and avidity [43] of LFA1. Deficiency in RAPL or Mst1 impairs chemokine-induced polarized cell shape changes and lymphocyte homing [90,91]. Mst1 and NDR1 are required for RhoA activation in T cells [92,93], suggesting that Rap1 signaling is involved in RhoA signaling. The localization of RAPL and Mst1 in vesicular compartments enriched for Rap1 and LFA1 suggests that they play a role in LFA1 transport involving Rab family proteins [89,94,95], thereby increasing LFA1 density and strengthening adhesion. Defective intracellular signaling through the Rap1-RAPL-Mst1/2-NDR1/2 axis mislocalizes vesicle transport regulators, and diminishes LFA1 in IS [35]. In addition, RAPL-associated Mst1 phosphorylates and activates NDR1 kinase. Activated NDR1 recruits kindlin-3 to the inner peripheral supramolecular activation cluster (pSMAC, see below) region, which is involved in high-affinity LFA1 binding to ICAM1 in IS [35]. The knockout or knockdown of each component of the Rap1-RAPL-Mst1/2 also impairs kindlin-3 localization in IS [35], suggesting that they act in the single genetic pathway.

## 4. Affinity Measurements of LFA1 and ICAM1 Interactions

Previous studies using surface plasmon resonance have revealed that the ICAM1-binding affinities of recombinant αI domains of LFA1 have three distinct classes: high (*K*_D_ of 150–360 nM), intermediate (*K*_D_ of 3–9 μM), and low (*K*_D_ of 0.5–1.6 mM) affinities [11], which are assumed to correspond to bent-closed, extended-closed, and extended-open conformations of LFA1, respectively. Upregulation of ICAM1 binding affinities of LFA1 on T cells, measured using soluble monomeric ICAM1, is modest: basal affinity (*K_D_*) is 36 μM in resting T cells, increasing to 23–29 μM in T cells stimulated with TCR crosslinking, SDF, or PMA, all of which can induce adhesion to immobilized ICAM1 [96]. These stimuli substantially induce an epitope that reports the extension (KIM127), with minimal impact on a βI activation reporter antibody epitope (mAb24) [96]. Thus, physiological activators of adhesion induce extension of LFA1 with only modest affinity changes for the soluble ligand. These findings indicate that inside-out signaling induces limited conformational and affinity changes for soluble ligands. Findings further suggest that only immobilized ligands can induce conformational changes to the extended-open conformation in a high-affinity state. An immobilized, but not soluble, ligand could accelerate conformational changes by exerting tension to ICAM1-bound LFA1 when LFA1 is linked to the F-actin cytoskeleton [96]. Alternatively, it is low-intermediate affinity LFA1 rather than high-affinity LFA1 that supports adhesion in concert with avidity changes and post-adhesion events.

## 5. Single-Molecule Measurements of Ligand Binding and Integrin Adaptor Proteins

Single-molecule imaging and the tracking of biomolecules in living cells have become increasingly powerful tools in providing real-time quantitative information about the kinetics, location, and dynamics of molecular interactions [97]. Conventional biochemical assays, including cell-based soluble ligand bindings, cell-lysate based pull-down assays, or those using surface plasmon resonance, are ensemble-averaging measurements and unable to clarify the working mechanisms of agonist-induced adhesive responses. To investigate how inside-out/outside-in signaling processes collaboratively regulate adhesion, we have introduced single-molecule measurements of LFA1 bindings to ICAM1 and integrin adaptors talin1 and kindlin-3.

### 5.1. LFA1 and ICAM1 Interactions

**Figure 3:**
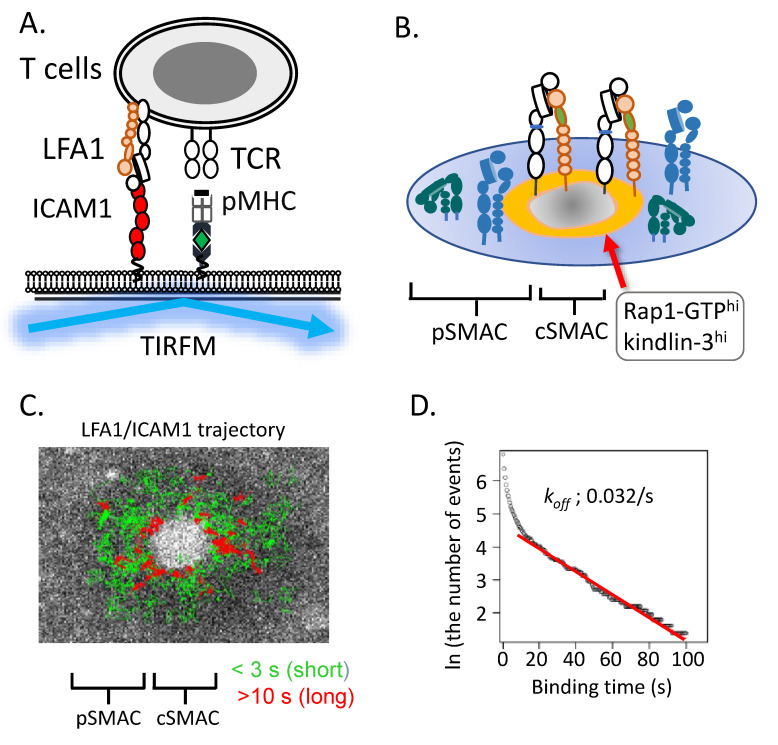
LFA1/ICAM1 interactions in immunological synapse. (**A**). Schematic representation of the experimental setting of single-molecule live imaging with TIRFM. Planar lipid bilayers incorporating fluorescently-labelled ICAM1 and peptide/MHC are used as surrogate antigen-presenting cells. (**B**). Immunological synapse with central SMAC (TCR/pMHC cluster) and pSMAC (LFA1/ICAM1). The inner pSMAC regions exhibiting high amounts of Rap1-GTP and kindlin-3 with high-affinity LFA1 are indicated. (**C**). Trajectories of LFA1-bound ICAM1 in IS. The trajectories with bound lifetime less than 3 s (green) and longer than 10 s (red) are shown [35]. (**D**). The semilogarithmic plot of LFA1/ICAM1 binding times and event numbers in IS. The slope (red) of the fitting line is *k_off_* (dissociation rate constant) of the stable binding longer than 10 s [35].

For the measurement of LFA1/ICAM1 interactions, we used immunological synapse reconstituted on planar lipid bilayers, incorporating peptide/MHC and labelled ICAM1-GPI, which have a high degree of Brownian motion. The mature synapse exhibits central SMAC (cSMAC) containing TCR/pMHC cluster with an outer ring of LFA1/ICAM1 (peripheral SMAC, pSMAC) [98]. ICAM1 was imaged in real-time at the single-molecule level by total internal reflection fluorescence microscopy (TIRFM) (Figure 3). Upon binding to LFA1, the lateral mobility of ICAM1 decreased. Single particle tracking (SPT) analysis of individual ICAM1 trajectories yielded diffusion coefficients, which were two orders smaller than those in cell-free areas [35]. Ensembled tracking data can provide an estimate of dissociation rate constants (*k*_off_) of LFA1–ICAM1 interactions. In the immunological synapse of primary OT-II T cells on planar lipid bilayers presenting ICAM1 and peptide/MHC, we found that half of LFA1–ICAM1 interactions were less than 1 s, whereas about 20–30% of interactions ranged from 1–3 s. These transient LFA1–ICAM1 interactions occurred in the pSMAC. Notably, a minor population (~10%) exhibited relatively stable LFA1 binding within the inner pSMAC near the central SMAC (cSMAC) [35]. The distribution of stable binding times was fitted to the first-order dissociation reaction, yielding *k*_off_ of 0.032 s^−1^ (average binding time of 31.2 s) with a high correlation (γ^2^ = 0.99) for binding times longer than 10 s. The calculated dissociation rate constant is consistent with those of high-affinity mutants of the αL I domain [11] or intact LFA1 [99]. The localization of stable ligand bindings in close proximity to the cSMAC agrees with the study of IS using activation reporter antibodies, showing that extended-open LFA1 conformations are exclusively localized in the inner pSMAC areas [100]. The stable bindings of LFA1 to ICAM1 lasting longer than 10 s are almost sessile (*D* = 1.9 × 10^−3^ μm^2^/s), suggesting the tethering of LFA1 to the cytoskeleton [35]. The low diffusion coefficients are comparable to those of low-mobility fractions of LFA1 increased upon cell attachment to ICAM1 [101]. Single-molecule measurements of LFA1/ICAM1 interactions, combined with other imaging and biochemical methods, demonstrate that: (1) there are at least two ligand binding affinity states of LFA1 in IS: a high-affinity state comparable to that of the open headpiece/extended conformation, and low/intermediate-affinity states, likely representing closed headpiece/extended conformers. The high-affinity state of LFA1 is relatively rare compared to low/intermediate states in mature IS. (2) The distribution of high-affinity ligand-bound LFA1 is restricted to the inner pSMAC zone with higher amounts of activated Rap1 and kindlin-3. (3) The localization of high-affinity binding requires Rap1 signaling, in which NDR1 associates with and recruits kindlin-3 to the inner pSMAC via RAPL/Mst1.

In line with the above findings, the IS on planar bilayers using human T lymphoblasts and anti-CD3 antibodies has exhibited a concentric distribution of outer extended LFA1 (KIM127^+^) with inner extended-open conformers (mAb24^+^) [100]. Thus, although interactions between LFA1 and ICAM1 are mediated predominantly by low/intermediate affinities of LFA1 that exist throughout the entire pSMAC, extended-open conformations with a high-affinity of LFA1 are generated in low frequencies in the specialized inner region of the pSMAC. The single-molecule measurement of ICAM1 in the naive T-cell IS has demonstrated that high-affinity LFA1 bound to ICAM1 is almost sessile (diffusion coefficients on the order of 10^−3^ μm^2^/s), and centripetal ICAM1 movement was not detected in naive T-cell IS, despite comparable frequencies of high-affinity LFA1 in T lymphoblasts [35]. These findings indicate that centripetal migration of engaged ICAM1 occurs in F-actin-rich T lymphoblasts and cells alike, which may not be essential for affinity maturation. Anchorage of LFA1 to the F-actin cytoskeleton via talin is sufficient for the generation of high-affinity binding, at least in naive T cells.

### 5.2. Single-Molecule Measurements of Integrin Adaptors to LFA1

Single-molecule imaging was applied to talin1 and kindlin-3 using a mouse lymphoid cell line Ba/F3 expressing human LFA1 (BAF/LFA1), which was engineered for single-molecule imaging through the deletion of endogenous talin1 or kindlin-3, and the introduction of halotag fusion proteins of talin1 or kindlin-3 (HT-Tln1, HT-Kin3). Endogenous β1, β2, β3, and β7 integrins were deleted to examine binding specificities of HT-Tln1 and HT-Kin3 for the human β2 tail [102]. BAF/LFA1 cells expressing HT-Tln1 or HT-Kin3 adhered to ICAM1 in response to PMA and CXCL12, which depend on talin1 and kindlin-3 expression. Dilute labeling of those cells with halotag ligands can visualize HT-Tln1 and HT-Kin3 at the single-molecule level near the plasma membrane by TIRFM. The SPT analysis of talin1 and kindlin-3 provides important information on their translocation frequencies, lifetime, and diffusion within the membrane, and binding kinetics to the β2 tail. In addition, the advantages of using human over mouse LFA1 include the availability of well-defined activating and reporter antibodies to human LFA1.

**Figure 4:**
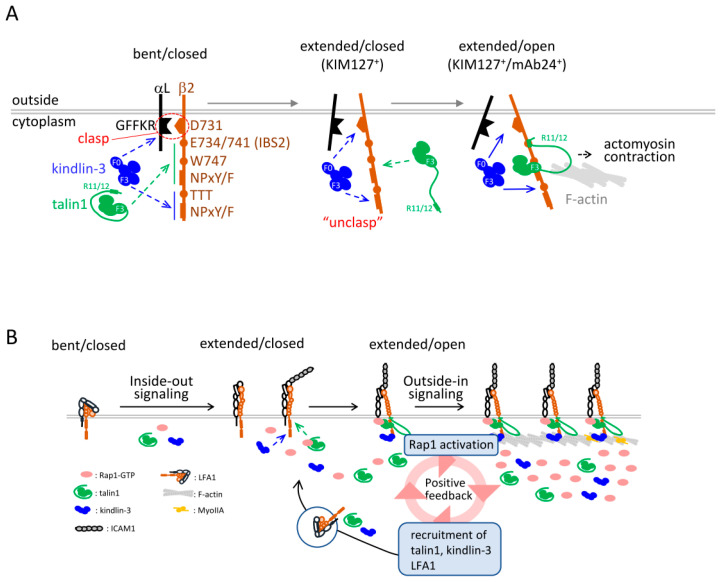
LFA1 activation processes. (**A**). Interactions of talin1 and kindlin-3 and cytoplasmic tails of LFA1. Critical residues in cytoplasmic tails that interact with talin1 and kindlin-3 are shown. Interactions of talin1 and kindlin-3 with the β2 tail infrequently occur with a lifetime less than 1 s. Stimulation with PMA or chemokines induce unbending (KIM127^+^), and tend to increase the number of sub-second interactions of talin1 and kindlin-3. ICAM1 triggers conformational changes (mAb24^+^) concomitant with increased longer bindings of talin1 and kindlin-3. Kindlin-3 loosens α/β clasps and stabilizes talin1 bindings to the β2 tail through IBS1 (F3), IBS2 (R11/12), and a linkage with actomyosin [102]. (**B**). Positive feedback of LFA1 activation. Once the extended-open headpiece with high-affinity ICAM1-bound LFA1 is formed, the outside-in signaling amplifies Rap1 signaling for the recruitment of talin1/kindlin-3 and LFA1 to growing attachment sites, thereby increasing the probability of co-occupancy with ICAM1 and integrin adaptors [102]. Adapted with permission from [102]. Copyright © 2022, AAAS.

The SPT analysis, combined with biochemical and functional studies, highlights the following regarding LFA1 activation: (1) **The mutual dependency of talin1 and kindlin-3.** Talin1 and kindlin-3 are mutually dependent for binding to the β2 tail in agonist-induced adhesion to ICAM1. (2) **The co-occupancy of LFA1 with ICAM1 and talin1**. The binding kinetics of talin1 to the β2 tail corresponded to those of LFA1 to ICAM1, whereas kindlin-3 has a shorter lifetime than talin1. (3) **The role of kindlin-3 in unclasping.** ICAM1 binding induced transient interactions between the F0 domain of kindlin-3 and the D731 in the salt bridge of the β2 tail, leading to unbending of LFA1 (KIM127^+^). (4) **The requirement of IBS2 and actomyosin.** IBS2 in the talin-R and actomyosin were required for high-affinity binding of talin1 to extended-open conformers (KIM127^+^mAb24^+^), but not to extended-closed conformers (KIM127^+^ mAb24^−^) of LFA1. (5) **The requirement of Rap1 and ICAM1 for the binding of talin1 and kindlin-3 to the β2 tail.** The absence of Rap1 or ICAM1 did not induce significant translocations of talin1 and kindlin-3. Overexpression of the activated Rap1 mutant was also insufficient for stable bindings of talin1 and kindlin-3 to the β2 tail, for which ICAM1 is necessary. (6) **Positive feedback amplification.** ICAM1 binding or stabilization of extended-open conformers (mAb24^+^) greatly augmented Rap1 activation, which, in turn, induced the translocation of talin1 and kindlin-3, as well as LFA1 on the adherent membrane (Figure 4).

### 5.3. Translocation and Binding Kinetics of Integrin Adaptors

The SPT analysis of mutant β2 tails demonstrates that the translocation of talin1 and kindlin-3 is mediated by specific binding to the β2 tail (Figure 4A). As expected, the W747 and proximal NPxY/F motif for talin1, and the TTT and distal NPxY/F motif for kindlin-3, were critical binding sites of the β2 tail. Translocation frequencies of talin1 and kindlin-3 to the membrane were greatly reduced by the mutation of these sites, indicating that talin translocation to the membrane largely represented the binding to the β2 tail. As kindlin-3 depended on talin1 and the talin1 binding site of W747, besides the distal NPxY/F motif and TTT for translocation, kindlin-3 would translocate in association with talin1, although their direct interaction has not yet been demonstrated.

The kinetics of talin1 binding to the β2 tail corresponded to those of LFA1 binding to ICAM1. The findings that the absence of talin1 abolished attachment to ICAM1, and that ICAM1 is required for talin1 translocation and binding, indicate the co-occupancy of LFA1 with ICAM1 and talin1. Notably, the dissociation rate constant of high-affinity talin1 binding to the β2 tail was comparable to that of high-affinity LFA1 (*k*_off_ talin1 vs. *k*_off_ LFA1-ICAM1: 0.0448 vs. 0.0524 s^−1^, average lifetime: 19.1 vs. 22.3 s). On the other hand, the lifetime of kindlin-3 (*k*_off_: 0.0975 s^−1^, average lifetime: 10.3 s) was two-fold faster than that of talin-1, indicating that kindlin-3 is unlikely to form a stable complex with talin1 and β2 tails. In line with the previous study [103], talin-H exhibits a short binding lifetime, predominantly less than 1 s, and weak adhesion-induced activity for LFA1, despite its translocation frequencies being comparable to those of full-length talin1. The additional interactions of IBS2 in talin-R (R11/R12) with conserved E734/741 of the β2 tail were required for high-affinity bindings of talin1. In addition, high-affinity talin1 exhibited low diffusion coefficients, in agreement with the finding that a linkage to actomyosin was necessary for high-affinity talin binding. These findings support the notion that a linkage of talin1 with actomyosin exerts tensile force on immobilized ICAM1-bound LFA1, generating high-affinity LFA1.

Translocation frequencies of both talin1 and kindlin-3 were severely reduced in the absence of Rap1. The deletion of the F0 domain of talin-H abolished translocation of talin1, in agreement with a previous report showing direct binding of Rap1-GTP to the F0 domain in vitro [46]. By contrast, the absence of RIAM had no impact on translocation and binding lifetime of talin1 to extended-open conformers of LFA1 in BAF/LFA1 cells, whereas agonist-induced adhesion to ICAM1 of BAF/LFA1 deficient in RIAM was defective, as was observed in lymphocytes [83]. Rap1 activation normally occurred in RIAM-deficient BAF/LFA1 cells. Thus, our SPT analysis did not identify any role of RIAM in talin dynamics, and the analysis supports the notion that activated Rap1 directly interacts with and recruits talin1 for association with the β tails. Kindlin-3 translocation also required activated Rap1; however, unlike the talin-H F0 domain, the kindlin-3 F0 domain does not interact with Rap1 [47], but binds to D731 in the β2 tail for unclasping, as shown in our study.

The high-affinity open conformers stabilized with mAb24 induced Rap1GEF C3G translocation to the membrane, which greatly augmented Rap1 activation and translocation of talin1 and kindlin-3. Single-molecule measurements of Rap1 show short dwell times in the plasma membrane: 1.27 s for wild-type and inactive Rap1, and 1.70 s for activated Rap1. Therefore, the membrane localization of activated Rap1 is due to a small difference in average dwell times between active and inactive Rap1. This suggests that Rap1 quickly transports talin1 and dissociates from the plasma membrane, and would not form a stable complex with talin1 bound to the β2 tail.

### 5.4. Coupling of Affinity and Avidity Modulation of LFA1

Integrins are known to accumulate on the contact membrane of adherent cells. ICAM1-mediated attachment increased LFA1 density on contact membranes of BAF/LFA1 cells approximately two-fold compared to those of non-adherent cells [102]. Stabilized extended-open conformations using immobilized mAb24 increased LFA1 density on the contact membrane approximately 4–5 times more than those using ICAM1. This was not observed with stabilized extended-closed conformations (KIM127^+^). Although conventional β2 antibodies (TS1/18) also increased LFA1 density about two-fold, translocation of integrin adaptors did not occur, implying a different mechanism of LFA1 recruitment by high-affinity conformers. Aside from lateral diffusion of LFA1, increased LFA1 on the contact membrane may occur via intracellular transport, as the Rap1 signaling is involved in the proper localization of vesicle transport regulators, as mentioned above. LFA1-containing vesicles increased with a reciprocal decrease in dorsal membranes in cells stimulated with activation reporter antibodies. Thus, the outside-in signaling through extended-open conformers transports LFA1 and integrin adaptors to the contact membrane, thereby further accelerating the association of integrin adaptors with the β2 tail and subsequent co-occupancy of integrin adaptors and ICAM1 (Figure 4B).

## 6. Summary

Single-molecule measurements of the bindings of ICAM1 and talin1/kindlin-3 with LFA1 have revealed bidirectional properties of LFA1 activation and adhesion. The findings of the single-particle tracking analysis suggest that LFA activation processes occur as follows (Figure 4): in the resting states, basal interactions of talin1 and kindlin-3 with LFA1 occur infrequently, with a lifetime of less than a second. LFA1 is predominantly in low-affinity states (bent-closed conformations). Inside-out activation through Rap1 tends to increase their sub-second translocation frequencies, but is not sufficient for stable bindings to the β2 tail in the absence of ICAM1. ICAM1 binding triggers substantial translocation of talin1 and kindlin-3. Kindlin-3 facilitates disruption of the inhibitory salt bridge so that IBS1 in talin1-H and IBS2 in talin-R can stabilize talin1 binding to the β2 tail, leading to separation of the α/β intersubunit association by actomyosin linked to talin-R. This transforms inactive bent LFA1 into fully activated extended/open LFA1 that transduce outside-in signaling through Rap1 activation, resulting in enhanced translocation of talin1 and kindlin-3, as well as LFA1. Thus, once rare high-affinity bindings with the extended-open headpiece are formed, the positive feedback circuit of Rap1 signaling is switched on for the recruitment of talin1/kindlin-3 and LFA1 to the attachment sites. As a result, the probability of co-occupancy of the neighboring LFA1 with ICAM1 and integrin adaptors is augmented, thereby increasing the amount of high-affinity LFA1, and leading to the establishment of a new equilibrium among bent-closed, extended-closed, and extended-open conformations in growing adhesion sites. The shift to a new equilibrium is, therefore, regulated by the coupling of affinity and avidity modulation of LFA1, and maintained by sustained Rap1 activation.

Though bidirectional LFA1 activation is tightly regulated, the bidirectionality of integrin activation can vary not only among different integrins, but also across different cellular and environmental conditions, the context of which provides important clues to find molecular targets for the development of therapeutics for immune diseases and integrin-involved pathological disorders.

## References

[1] Hanna S., Etzioni A. (2012). Leukocyte adhesion deficiencies. Ann. N. Y. Acad. Sci..

[2] Hogg N., Patzak I., Willenbrock F. (2011). The insider’s guide to leukocyte integrin signalling and function. Nat. Rev. Immunol..

[3] Dustin M., Springer T.A. (1989). T-cell receptor cross-linking transiently stimulates adhesiveness through LFA-1. Nature.

[4] Kinashi T. (2005). Intracellular signalling controlling integrin activation in lymphocytes. Nat. Rev. Immunol..

[5] Moser M., Legate K.R., Zent R., Fassler R. (2009). The tail of integrins, talin, and kindlins. Science.

[6] Calderwood D.A., Campbell I.D., Critchley D.R. (2013). Talins and kindlins: Partners in integrin-mediated adhesion. Nat. Rev. Mol. Cell Biol..

[7] Rognoni E., Ruppert R., Fässler R. (2016). The kindlin family: Functions, signaling properties and implications for human disease. J. Cell Sci..

[8] Takagi J., Springer T.A. (2002). Integrin activation and structural rearrangement. Immunol. Rev..

[9] Luo B.-H., Carman C.V., Springer T.A. (2007). Structural Basis of Integrin Regulation and Signaling. Annu. Rev. Immunol..

[10] Arnaout M.A. (2016). Biology and structure of leukocyte β2 integrins and their role in inflammation. F1000Research.

[11] Shimaoka M., Xiao T., Liu J.-H., Yang Y., Dong Y., Jun C.-D., McCormack A., Zhang R., Joachimiak A., Takagi J. (2003). Structures of the αL I Domain and Its Complex with ICAM-1 Reveal a Shape-Shifting Pathway for Integrin Regulation. Cell.

[12] Springer T.A. (1995). Traffic signals on endothelium for lymphocyte recirculation and leukocyte emigration. Annu. Rev. Physiol..

[13] Lebedeva T., Dustin M., Sykulev Y. (2005). ICAM-1 co-stimulates target cells to facilitate antigen presentation. Curr. Opin. Immunol..

[14] Xiong J.P., Stehle T., Zhang R., Joachimiak A., Frech M., Goodman S.L., Arnaout M.A. (2002). Crystal structure of the extracellular segment of integrin alpha Vbeta3 in complex with an Arg-Gly-Asp ligand. Science.

[15] Adair B.D., Xiong J.-P., Maddock C., Goodman S.L., Arnaout M.A., Yeager M. (2005). Three-dimensional EM structure of the ectodomain of integrin αVβ3 in a complex with fibronectin. J. Cell Biol..

[16] Yu Y., Zhu J., Mi L.-Z., Walz T., Sun H., Chen J., Springer T.A. (2012). Structural specializations of α4β7, an integrin that mediates rolling adhesion. J. Cell Biol..

[17] Mould A.P., Symonds E.J.H., Buckley P.A., Grossmann J.G., McEwan P.A., Barton S.J., Askari J.A., Craig S.E., Bella J., Humphries M. (2003). Structure of an Integrin-Ligand Complex Deduced from Solution X-ray Scattering and Site-directed Mutagenesis. J. Biol. Chem..

[18] Chen J., Salas A., A Springer T. (2003). Bistable regulation of integrin adhesiveness by a bipolar metal ion cluster. Nat. Struct. Mol. Biol..

[19] Takagi J., Petre B.M., Walz T., Springer T.A. (2002). Global Conformational Rearrangements in Integrin Extracellular Domains in Outside-In and Inside-Out Signaling. Cell.

[20] Nishida N., Xie C., Shimaoka M., Cheng Y., Walz T., Springer T.A. (2006). Activation of Leukocyte β2 Integrins by Conversion from Bent to Extended Conformations. Immunity.

[21] Sen M., Koksal A., Yuki K., Wang J., Springer T.A. (2018). Ligand- and cation-induced structural alterations of the leukocyte integrin LFA-1. J. Biol. Chem..

[22] Li J., Su Y., Xia W., Qin Y., Humphries M., Vestweber D., Cabañas C., Lu C., A Springer T. (2017). Conformational equilibria and intrinsic affinities define integrin activation. EMBO J..

[23] Li J., Springer T.A. (2017). Energy landscape differences among integrins establish the framework for understanding activation. J. Cell Biol..

[24] Wegener K.L., Campbell I.D. (2008). Transmembrane and cytoplasmic domains in integrin activation and protein-protein interactions (Review). Mol. Membr. Biol..

[25] Lu C.F., A Springer T. (1997). The alpha subunit cytoplasmic domain regulates the assembly and adhesiveness of integrin lymphocyte function-associated antigen-1. J. Immunol..

[26] Kim M., Carman C.V., Springer T.A. (2003). Bidirectional Transmembrane Signaling by Cytoplasmic Domain Separation in Integrins. Science.

[27] Chigaev A., Buranda T., Dwyer D.C., Prossnitz E.R., Sklar L.A. (2003). FRET Detection of Cellular α4-Integrin Conformational Activation. Biophys. J..

[28] Ye F., Hu G., Taylor D., Ratnikov B., Bobkov A.A., McLean M.A., Sligar S.G., Taylor K.A., Ginsberg M.H. (2010). Recreation of the terminal events in physiological integrin activation. J. Cell Biol..

[29] Lu C., Ferzly M., Takagi J., Springer T.A. (2001). Epitope mapping of antibodies to the C-terminal region of the integrin beta 2 subunit reveals regions that become exposed upon receptor activation. J. Immunol..

[30] Fan Z., McArdle S., Mark G., Mikulski Z., Gutierrez E., Engelhardt B., Deutsch U., Ginsberg M., Groisman A., Ley K. (2016). Neutrophil recruitment limited by high-affinity bent β2 integrin binding ligand in cis. Nat. Commun..

[31] Fan Z., Kiosses W.B., Sun H., Orecchioni M., Ghosheh Y., Zajonc D.M., Arnaout M.A., Gutierrez E., Groisman A., Ginsberg M.H. (2019). High-Affinity Bent β2-Integrin Molecules in Arresting Neutrophils Face Each Other through Binding to ICAMs In cis. Cell Rep..

[32] Bos J.L., De Rooij J., Reedquist K.A. (2001). Rap1 signalling: Adhering to new models. Nat. Rev. Mol. Cell Biol..

[33] Chrzanowska-Wodnicka M., Smyth S.S., Schoenwaelder S.M., Fischer T.H., White G.C. (2005). Rap1b is required for normal platelet function and hemostasis in mice. J. Clin. Investig..

[34] Chu H., Awasthi A., White G.C., Chrzanowska-Wodnicka M., Malarkannan S. (2008). Rap1b Regulates B Cell Development, Homing, and T Cell-Dependent Humoral Immunity. J. Immunol..

[35] Kondo N., Ueda Y., Kita T., Ozawa M., Tomiyama T., Yasuda K., Lim D.-S., Kinashi T. (2017). NDR1-Dependent Regulation of Kindlin-3 Controls High-Affinity LFA-1 Binding and Immune Synapse Organization. Mol. Cell. Biol..

[36] Ishihara S., Nishikimi A., Umemoto E., Miyasaka M., Saegusa M., Katagiri K. (2015). Dual functions of Rap1 are crucial for T-cell homeostasis and prevention of spontaneous colitis. Nat. Commun..

[37] Su W., Wynne J., Pinheiro E.M., Strazza M., Mor A., Montenont E., Berger J., Paul D.S., Bergmeier W., Gertler F.B. (2015). Rap1 and its effector RIAM are required for lymphocyte trafficking. Blood.

[38] Ueda Y., Kondo N., Ozawa M., Yasuda K., Tomiyama T., Kinashi T. (2016). Sema3e/Plexin D1 Modulates Immunological Synapse and Migration of Thymocytes by Rap1 Inhibition. J. Immunol..

[39] Crittenden J.R., Bergmeier W., Zhang Y., Piffath C.L., Liang Y., Wagner D.D., Housman D.E., Graybiel A.M. (2004). CalDAG-GEFI integrates signaling for platelet aggregation and thrombus formation. Nat. Med..

[40] Reedquist K.A., Ross E., Koop E.A., Wolthuis R., Zwartkruis F.J., Van Kooyk Y., Salmon M., Buckley C.D., Bos J.L. (2000). The Small Gtpase, Rap1, Mediates Cd31-Induced Integrin Adhesion. J. Cell Biol..

[41] Azoulay-Alfaguter I., Strazza M., Peled M., Novak H.K., Muller J., Dustin M.L., Mor A. (2017). The tyrosine phosphatase SHP-1 promotes T cell adhesion by activating the adaptor protein CrkII in the immunological synapse. Sci. Signal..

[42] Stefanini L., Bergmeier W. (2016). RAP1-GTPase signaling and platelet function. J. Mol. Med..

[43] Katagiri K., Maeda A., Shimonaka M., Kinashi T. (2003). RAPL, a Rap1-binding molecule that mediates Rap1-induced adhesion through spatial regulation of LFA-1. Nat. Immunol..

[44] Lafuente E.M., van Puijenbroek A.A., Krause M., Carman C.V., Freeman G.J., Berezovskaya A., Constantine E., Springer T.A., Gertler F.B., Boussiotis V.A. (2004). RIAM, an Ena/VASP and Profilin Ligand, Interacts with Rap1-GTP and Mediates Rap1-Induced Adhesion. Dev. Cell.

[45] Plak K., Pots H., Van Haastert P.J.M., Kortholt A. (2016). Direct Interaction between TalinB and Rap1 is necessary for adhesion of Dictyostelium cells. BMC Cell Biol..

[46] Zhu L., Yang J., Bromberger T., Holly A., Lu F., Liu H., Sun K., Klapproth S., Hirbawi J., Byzova T. (2017). Structure of Rap1b bound to talin reveals a pathway for triggering integrin activation. Nat. Commun..

[47] Bromberger T., Zhu L., Klapproth S., Qin J., Moser M. (2019). Rap1 and membrane lipids cooperatively recruit talin to trigger integrin activation. J. Cell Sci..

[48] Bromberger T., Klapproth S., Rohwedder I., Zhu L., Mittmann L., Reichel C.A., Sperandio M., Qin J., Moser M. (2018). Direct Rap1/Talin1 interaction regulates platelet and neutrophil integrin activity in mice. Blood.

[49] Lagarrigue F., Gingras A.R., Paul D.S., Valadez A.J., Cuevas M.N., Sun H., Lopez-Ramirez M.A., Goult B.T., Shattil S.J., Bergmeier W. (2018). Rap1 binding to the talin 1 F0 domain makes a minimal contribution to murine platelet GPIIb-IIIa activation. Blood Adv..

[50] Tadokoro S., Shattil S.J., Eto K., Tai V., Liddington R.C., de Pereda J.M., Ginsberg M.H., Calderwood D.A. (2003). Talin binding to integrin beta tails: A final common step in integrin activation. Science.

[51] Shattil S.J., Kim C., Ginsberg M.H. (2010). The final steps of integrin activation: The end game. Nat. Rev. Mol. Cell Biol..

[52] Nieswandt B., Moser M., Pleines I., Varga-Szabo D., Monkley S., Critchley D., Fassler R. (2007). Loss of talin1 in platelets abrogates integrin activation, platelet aggregation, and thrombus formation in vitro and in vivo. J. Exp. Med..

[53] Wernimont S.A., Wiemer A.J., Bennin D.A., Monkley S.J., Ludwig T., Critchley D.R., Huttenlocher A. (2011). Contact-Dependent T Cell Activation and T Cell Stopping Require Talin1. J. Immunol..

[54] Calderwood D.A., Zent R., Grant R., Rees D.J., Hynes R.O., Ginsberg M.H. (1999). The talin head domain binds to integrin beta subunit cytoplasmic tails and regulates integrin activation. J. Biol. Chem..

[55] Anthis N.J., Wegener K.L., Ye F., Kim C., Goult B.T., Lowe E.D., Vakonakis I., Bate N., Critchley D.R., Ginsberg M.H. (2009). The structure of an integrin/talin complex reveals the basis of inside-out signal transduction. EMBO J..

[56] Rodius S., Chaloin O., Moes M., Schaffner-Reckinger E., Landrieu I., Lippens G., Lin M., Zhang J., Kieffer N. (2008). The Talin Rod IBS2 α-Helix Interacts with the β3 Integrin Cytoplasmic Tail Membrane-proximal Helix by Establishing Charge Complementary Salt Bridges. J. Biol. Chem..

[57] Goksoy E., Ma Y.-Q., Wang X., Kong X., Perera D., Plow E.F., Qin J. (2008). Structural Basis for the Autoinhibition of Talin in Regulating Integrin Activation. Mol. Cell.

[58] Goult B.T., Xu X.-P., Gingras A.R., Swift M., Patel B., Bate N., Kopp P.M., Barsukov I.L., Critchley D.R., Volkmann N. (2013). Structural studies on full-length talin1 reveal a compact auto-inhibited dimer: Implications for talin activation. J. Struct. Biol..

[59] Song X., Yang J., Hirbawi J., Ye S., Perera H.D., Goksoy E., Dwivedi P., Plow E.F., Zhang R., Qin J. (2012). A novel membrane-dependent on/off switch mechanism of talin FERM domain at sites of cell adhesion. Cell Res..

[60] Legate K.R., Takahashi S., Bonakdar N., Fabry B., Boettiger D., Zent R., Fässler R. (2011). Integrin adhesion and force coupling are independently regulated by localized PtdIns(4,5)2synthesis. EMBO J..

[61] Yago T., Zhang N., Zhao L., Abrams C.S., McEver R.P. (2018). Selectins and chemokines use shared and distinct signals to activate β2 integrins in neutrophils. Blood Adv..

[62] Moser M., Nieswandt B., Ussar S., Pozgajova M., Fässler R. (2008). Kindlin-3 is essential for integrin activation and platelet aggregation. Nat. Med..

[63] Malinin N.L., Zhang L., Choi J., Ciocea A., Razorenova O., Ma Y.-Q., A Podrez E., Tosi M., Lennon D.P., I Caplan A. (2009). A point mutation in KINDLIN3 ablates activation of three integrin subfamilies in humans. Nat. Med..

[64] Moser M., Bauer M., Schmid S., Ruppert R., Schmidt S., Sixt M., Wang H.V., Sperandio M., Fassler R. (2009). Kindlin-3 is required for beta2 integrin-mediated leukocyte adhesion to endothelial cells. Nat. Med..

[65] Svensson L., Howarth K., McDowall A., Patzak I., Evans R., Ussar S., Moser M., Metin A., Fried M., Tomlinson I. (2009). Leukocyte adhesion deficiency-III is caused by mutations in KINDLIN3 affecting integrin activation. Nat. Med..

[66] Lefort C.T., Ley K. (2012). Neutrophil arrest by LFA-1 activation. Front. Immunol..

[67] Bledzka K., Liu J., Xu Z., Perera H.D., Yadav S.P., Bialkowska K., Qin J., Ma Y.-Q., Plow E.F. (2012). Spatial Coordination of Kindlin-2 with Talin Head Domain in Interaction with Integrin β Cytoplasmic Tails. J. Biol. Chem..

[68] Hart R., Stanley P., Chakravarty P., Hogg N. (2013). The Kindlin 3 Pleckstrin Homology Domain Has an Essential Role in Lymphocyte Function-associated Antigen 1 (LFA-1) Integrin-mediated B Cell Adhesion and Migration. J. Biol. Chem..

[69] Wen L., Marki A., Roy P., McArdle S., Sun H., Fan Z., Gingras A.R., Ginsberg M.H., Ley K. (2021). Kindlin-3 recruitment to the plasma membrane precedes high-affinity β2-integrin and neutrophil arrest from rolling. Blood.

[70] Theodosiou M., Widmaier M., Böttcher R.T., Rognoni E., Veelders M., Bharadwaj M., Lambacher A., Austen K., Muller D.J., Zent R. (2016). Kindlin-2 cooperates with talin to activate integrins and induces cell spreading by directly binding paxillin. eLife.

[71] Nguyen H.T.T., Xu Z., Shi X., Liu S., Schulte M.L., White G.C., Ma Y. (2021). Paxillin binding to the PH domain of kindlin-3 in platelets is required to support integrin αIIbβ3 outside-in signaling. J. Thromb. Haemost..

[72] Böttcher R.T., Böttcher R.T., Veelders M., Veelders M., Rombaut P., Rombaut P., Faix J., Faix J., Theodosiou M., Theodosiou M. (2017). Kindlin-2 recruits paxillin and Arp2/3 to promote membrane protrusions during initial cell spreading. J. Cell Biol..

[73] Gao J., Huang M., Lai J., Mao K., Sun P., Cao Z., Hu Y., Zhang Y., Schulte M.L., Jin C. (2017). Kindlin supports platelet integrin αIIbβ3 activation by interacting with paxillin. J. Cell Sci..

[74] Zhu L., Liu H., Lu F., Yang J., Byzova T.V., Qin J. (2019). Structural Basis of Paxillin Recruitment by Kindlin-2 in Regulating Cell Adhesion. Structure.

[75] Bledzka K., Bialkowska K., Sossey-Alaoui K., Vaynberg J., Pluskota E., Qin J., Plow E.F. (2016). Kindlin-2 directly binds actin and regulates integrin outside-in signaling. J. Cell Biol..

[76] Ye F., Petrich B.G., Anekal P., Lefort C.T., Kasirer-Friede A., Shattil S.J., Ruppert R., Moser M., Fässler R., Ginsberg M.H. (2013). The Mechanism of Kindlin-Mediated Activation of Integrin alphaIIbbeta3. Curr. Biol..

[77] Li H., Deng Y., Sun K., Yang H., Liu J., Wang M., Zhang Z., Lin J., Wu C., Wei Z. (2017). Structural basis of kindlin-mediated integrin recognition and activation. Proc. Natl. Acad. Sci. USA.

[78] Sun J., Xiao D., Ni Y., Zhang T., Cao Z., Xu Z., Nguyen H., Zhang J., White G.C., Ding J. (2020). Structure basis of the FERM domain of kindlin-3 in supporting integrin αIIbβ3 activation in platelets. Blood Adv..

[79] Bu W., Levitskaya Z., Loh Z.Y., Jin S., Basu S., Ero R., Yan X., Wang M., Ngan S.F.C., Sze S.K. (2020). Structural basis of human full-length kindlin-3 homotrimer in an auto-inhibited state. PLoS Biol..

[80] Wynne J.P., Wu J., Su W., Mor A., Patsoukis N., Boussiotis V.A., Hubbard S.R., Philips M.R. (2012). Rap1-interacting adapter molecule (RIAM) associates with the plasma membrane via a proximity detector. J. Cell Biol..

[81] Yang J., Zhu L., Zhang H., Hirbawi J., Fukuda K., Dwivedi P., Liu J., Byzova T., Plow E.F., Wu J. (2014). Conformational activation of talin by RIAM triggers integrin-mediated cell adhesion. Nat. Commun..

[82] Lagarrigue F., Kim C., Ginsberg M.H. (2016). The Rap1-RIAM-talin axis of integrin activation and blood cell function. Blood.

[83] Klapproth S., Sperandio M., Pinheiro E.M., Prünster M., Soehnlein O., Gertler F.B., Fässler R., Moser M. (2015). Loss of the Rap1 effector RIAM results in leukocyte adhesion deficiency due to impaired β2 integrin function in mice. Blood.

[84] Stritt S., Wolf K., Lorenz V., Vögtle T., Gupta S., Bösl M.R., Nieswandt B. (2015). Rap1-GTP–interacting adaptor molecule (RIAM) is dispensable for platelet integrin activation and function in mice. Blood.

[85] Sun H., Lagarrigue F., Wang H., Fan Z., Lopez-Ramirez M.A., Chang J.T., Ginsberg M.H. (2020). Distinct integrin activation pathways for effector and regulatory T cell trafficking and function. J. Exp. Med..

[86] Krause M., Leslie J.D., Stewart M., Lafuente E.M., Valderrama F., Jagannathan R., Strasser G.A., Rubinson D.A., Liu H., Way M. (2004). Lamellipodin, an Ena/VASP Ligand, Is Implicated in the Regulation of Lamellipodial Dynamics. Dev. Cell.

[87] Law A.-L., Vehlow A., Kotini M., Dodgson L., Soong D., Theveneau E., Bodo C., Taylor E., Navarro C., Perera U. (2013). Lamellipodin and the Scar/WAVE complex cooperate to promote cell migration in vivo. J. Cell Biol..

[88] Richter A.M., Pfeifer G.P., Dammann R.H. (2009). The RASSF proteins in cancer; from epigenetic silencing to functional characterization. Biochim. Biophys. Acta.

[89] Katagiri K., Imamura M., Kinashi T. (2006). Spatiotemporal regulation of the kinase Mst1 by binding protein RAPL is critical for lymphocyte polarity and adhesion. Nat. Immunol..

[90] Katagiri K., Ohnishi N., Kabashima K., Iyoda T., Takeda N., Shinkai Y., Inaba K., Kinashi T. (2004). Crucial functions of the Rap1 effector molecule RAPL in lymphocyte and dendritic cell trafficking. Nat. Immunol..

[91] Katagiri K., Katakai T., Ebisuno Y., Ueda Y., Okada T., Kinashi T. (2009). Mst1 controls lymphocyte trafficking and interstitial motility within lymph nodes. EMBO J..

[92] Mou F., Praskova M., Xia F., Van Buren D., Hock H., Avruch J., Zhou D. (2012). The Mst1 and Mst2 kinases control activation of rho family GTPases and thymic egress of mature thymocytes. J. Exp. Med..

[93] Tang F., Gill J., Ficht X., Barthlott T., Cornils H., Schmitz-Rohmer D., Hynx D., Zhou D., Zhang L., Xue G. (2015). The kinases NDR1/2 act downstream of the Hippo homolog MST1 to mediate both egress of thymocytes from the thymus and lymphocyte motility. Sci. Signal..

[94] Nishikimi A., Ishihara S., Ozawa M., Etoh K., Fukuda M., Kinashi T., Katagiri K. (2014). Rab13 acts downstream of the kinase Mst1 to deliver the integrin LFA-1 to the cell surface for lymphocyte trafficking. Sci. Signal..

[95] Capece T., Walling B.L., Lim K., Kim K.-D., Bae S., Chung H.-L., Topham D.J., Kim M. (2017). A novel intracellular pool of LFA-1 is critical for asymmetric CD8+ T cell activation and differentiation. J. Cell Biol..

[96] Schürpf T., A Springer T. (2011). Regulation of integrin affinity on cell surfaces. EMBO J..

[97] Shen H., Tauzin L.J., Baiyasi R., Wang W., Moringo N., Shuang B., Landes C.F. (2017). Single Particle Tracking: From Theory to Biophysical Applications. Chem. Rev..

[98] Grakoui A., Bromley S.K., Sumen C., Davis M.M., Shaw A.S., Allen P.M., Dustin M.L. (1999). The Immunological Synapse: A Molecular Machine Controlling T Cell Activation. Science.

[99] E Labadia M., Jeanfavre D.D., O Caviness G., Morelock M.M. (1998). Molecular regulation of the interaction between leukocyte function-associated antigen-1 and soluble ICAM-1 by divalent metal cations. J. Immunol..

[100] Comrie W.A., Babich A., Burkhardt J.K. (2015). F-actin flow drives affinity maturation and spatial organization of LFA-1 at the immunological synapse. J. Cell Biol..

[101] Cairo C.W., Mirchev R., Golan D.E. (2006). Cytoskeletal Regulation Couples LFA-1 Conformational Changes to Receptor Lateral Mobility and Clustering. Immunity.

[102] Kondo N., Ueda Y., Kinashi T. (2021). Kindlin-3 disrupts an intersubunit association in the integrin LFA1 to trigger positive feedback activation by Rap1 and talin1. Sci. Signal..

[103] Anthis N.J., Wegener K.L., Critchley D.R., Campbell I.D. (2010). Structural Diversity in Integrin/Talin Interactions. Structure.

